# Jian-Pi-Yi-Shen Formula Regulates Inflammatory Cytokines Production in 5/6 Nephrectomized Rats via Suppression of NF-κB Activation

**DOI:** 10.1155/2018/7203547

**Published:** 2018-07-17

**Authors:** Jiandong Lu, Xinhui Liu, Yijiao Liao, Dongcai Wang, Jianping Chen, Shunmin Li

**Affiliations:** ^1^Department of Nephrology, Shenzhen Traditional Chinese Medicine Hospital, Guangzhou University of Chinese Medicine, Shenzhen, Guangdong, China; ^2^Centers for Disease Early Treatment, Shenzhen Traditional Chinese Medicine Hospital, Guangzhou University of Chinese Medicine, Shenzhen, Guangdong, China; ^3^Shenzhen Key Laboratory of Hospital Chinese Medicine Preparation, Shenzhen Traditional Chinese Medicine Hospital, Guangzhou University of Chinese Medicine, Shenzhen, Guangdong, China

## Abstract

Jian-Pi-Yi-Shen formula (JPYSF) is a Chinese herbal decoction used for treating chronic kidney disease (CKD) for over 20 years with good efficiency. However, the mechanism lacks solid evidence. In the present study, we tested the hypothesis that JPYSF may retard CKD progression via inhibition of inflammation in 5/6 nephrectomy (5/6 Nx) rat model. The 5/6 Nx rats were randomly divided into 2 groups: 5/6 Nx group and JPYSF group. Sham-operated rats served as control. JPYSF (2.06 g/kg/d) were administrated by gavage to 5/6 Nx rats daily for 6 weeks. Results showed that JPYSF treatment significantly improved kidney function and pathological injury in 5/6 Nx rats. Multiplex analysis of cytokines revealed that JPYSF reduced proinflammatory cytokines and increased anti-inflammatory cytokine production. Furthermore, JPYSF inhibited the activation of nuclear factor-kappa B (NF-*κ*B) signaling pathway. In conclusion, our data demonstrated that JPYSF remarkably retards development and progression of CKD in a 5/6 Nx rat model, which may be associated with inhibition of inflammation via NF-*κ*B signaling pathway.

## 1. Introduction

Chronic kidney disease (CKD) is characterized by persisting damage of renal structure and function and is an increasing public health issue [1]. Its prevalence is estimated to be 8-16% worldwide [2]. Unfortunately, there are relatively few therapies in development for the treatment of CKD [3]. Alternatively, patients with CKD in China and other Asian countries usually seek remedies in traditional Chinese medicine (TCM) [4, 5]. Recent studies showed that prescribed Chinese herbal medicines were associated with reduced risk of end-stage renal disease and mortality in patients with CKD [6, 7]. Jian-Pi-Yi-Shen formula (JPYSF) is a traditional Chinese herbal decoction and has been prescribed as a basic formula to CKD patients with good efficacy [8]. However, the underlying mechanisms of this efficacy remain unclear.

Inflammation plays a pivotal role in the development and progression of CKD. It has been reported that prevalence of inflammation varies from 30 to 75% in CKD patients [9]. Multiple factors can contribute to inflammation activation in CKD, such as oxidative stress [10], translocation of gut bacteria and bacterial components [11], metabolic acidosis [12], and vitamin D deficiency [13]. Consequently, persistent inflammation results in adverse cardiovascular outcomes [14], malnutrition/protein-energy wasting [15], anemia [16, 17], and mineral and bone disease [18, 19]. Therefore, targeting inflammation will be an effective therapeutic approach in CKD. The nuclear factor-kappa B (NF-*κ*B) signaling pathway has a key role in promoting transcription of proinflammatory genes and triggering inflammation cascade in a variety of inflammatory diseases [20]. Previous study showed that JPYSF could regulate the expression of proinflammatory cytokines in cultured macrophages, indicating that JPYSF may have anti-inflammation effect [21]. In the present study, we extend our effect in exploring the role of JPYSF on inflammation in 5/6 nephrectomy (5/6 Nx)-induced CKD model and the possible role of NF-*κ*B signaling pathway in this process.

## 2. Materials and Methods

### 2.1. Preparation of JPYSF Water Extract

Raw herbs were purchased from Shenzhen Huahui Pharmaceutical Co., Ltd. (Shenzhen, China) and were authenticated by Shangbin Zhang based on their morphological characteristics. Preparation procedures of JPYSF extract were conducted as previously described [21]. In brief, Astragali Radix (30 g), Atractylodis Macrocephalae Rhizoma (10 g), Dioscoreae Rhizoma (30 g), Cistanches Herba (10 g), Amomi Fructus Rotundus (10 g), Salviae Miltiorrhizae Radix et Rhizoma (15 g), Rhei Radix et Rhizoma (10 g), and Glycyrrhizae Radix et Rhizoma Praeparata cum Melle (6 g) were weighed and boiled twice in 8 times of ddH_2_O (w/v) for 1 hour per time. For animal studies, the extract was dried using freeze dryer and stored at -80°C. Before the treatment, the freeze-dried powder was redissolved with ddH_2_O to obtain JPYSF water extract.

### 2.2. Animals and Experimental Design

All animal experiments were conducted with protocols approved by the Ethics Committee of Shenzhen Traditional Chinese Medicine Hospital, Guangzhou University of Traditional Chinese Medicine (Shenzhen, China). Thirty male Sprague-Dawley rats weighing 180-220 g were purchased from Guangdong Medical Laboratory Animal Center (Foshan, China) and maintained in a specific pathogen-free animal facility under a 12-hour light/12-hour dark cycle, with free access to food and water. The 5/6 Nx operation was performed in rats under anesthesia with sodium pentobarbital (50 mg/kg body weight, intraperitoneal injection) by ablation of upper and lower thirds of the left kidney and then removal of the right kidney 2 weeks later. The sham operation consisting of laparotomy and manipulation of the renal pedicles but without destruction of renal tissue was performed. Twelve weeks after the second surgery, all rats were divided into 3 groups: the sham group, 5/6 Nx group, and JPYSF group. JPYSF extract was administrated by gavage daily at the dose of 2.06 g/kg. The same volume of distilled water was given to the sham and 5/6 Nx group. After 6 weeks of treatment, all rats were anesthetized (sodium pentobarbital, 50 mg/kg body weight, intraperitoneal injection), and blood samples were collected. Kidneys were removed and preserved for further analysis.

### 2.3. Biochemical Analysis

Serum creatinine (Scr) and blood urea nitrogen (BUN) were measured using BS-180 automatic biochemistry analyzer (Mindray, Shenzhen, China) following the manufacturer's instructions.

### 2.4. Histology

Periodic acid-Schiff (PAS) and Masson's trichrome stains were performed to evaluate the pathological changes of kidney. For quantitative analysis, tubular atrophy score in PAS staining was defined as follows: 0, normal tubules; 1, rare single atrophic tubule; 2, several clusters of atrophic tubules; 3, massive atrophy [22]. The fibrotic area in Masson staining was measured using Image J software (NIH, Bethesda, MD, USA). At least 10 microscopic fields (200×) of each rat and six rats in each group were performed atrophy score and fibrotic area measurement in a blinded manner.

### 2.5. Inflammatory Mediators

We selected a panel of cytokines and chemotactic cytokines to reflect various aspects of the immune-inflammatory system. Proinflammatory cytokines interleukin-1*β* (IL-*β*) and IL-6, anti-inflammatory cytokine IL-10, and chemotactic cytokines monocyte chemoattractant protein-1 (MCP-1), macrophage inflammatory protein-1*α* (MIP-1*α*), and MIP-2 were determined. The levels of inflammatory mediators in kidney were simultaneously analyzed by bead-based Milliplex (Millipore, St. Charles, MO, USA), according to the provided manufacturers' protocol [23].

### 2.6. Immunohistochemistry

The paraffin-embedded kidney slides were treated step-by-step by dewaxed, rehydrated, and antigen retrieval. Then the slides were incubated with 3% hydrogen peroxide for 10 minutes at room temperature and were blocked with 10% goat serum for 1 hour at 37°C. The sections were stained with p65 (1 : 100), p-p65 (Ser 536) (1 : 50), I*κ*B*α* (1 : 100), and p-I*κ*B*α* (Ser 32) (1 : 50) primary antibody (Cell Signaling Technology, Beverly, MA, USA) at 4°C overnight followed by SignalStain Boost Detection Reagent (Cell Signaling Technology, Beverly, MA, USA) for 30 min at room temperature. The sections were then treated with SignalStain diaminobenzidine (DAB) substrate (Cell Signaling Technology, Beverly, MA, USA), followed by counterstaining with hematoxylin and mounting. The Image-Pro Plus 6.0 software (Media Cybernetics, CA, USA) was used to calculate integrated optical density (IOD) values.

### 2.7. Statistical Analysis

Data are presented as mean ± SEM. One-way ANOVA was used to test statistical significance among groups followed by* post hoc* analysis using Least Significant Difference (LSD) test or Games-Howell test. Statistical significance was set at* P* value <0.05. All statistical analyses were performed using SPSS statistics software (version 16.0, SPSS Inc., Chicago, IL, USA).

## 3. Results and Discussion

### 3.1. JPYSF Improved Kidney Function in 5/6 Nx Rats

**Figure 1:**
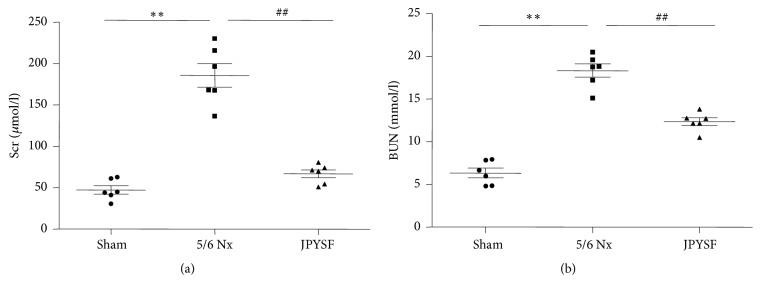
**JPYSF improved kidney function in 5/6 Nx rats.** (a) Scr levels. (b) BUN levels. Data are presented as the means ± SEM, n=6 rats per group (^*∗∗*^*P*<0.01 compared with the sham group; ^##^*P*<0.01 compared with the 5/6 Nx group).

As shown in Figure 1, Scr and BUN levels in 5/6 Nx rats were significantly elevated compared with the sham group (*P*<0.01). After administration of JPYSF for 6 weeks, both Scr and BUN levels were decreased (*P*<0.01). Impaired kidney function is the characteristic of CKD and is usually evaluated by Scr and BUN levels. This result indicated that CKD model had been successfully established by 5/6 Nx and JPYSF could retard CKD progression.

### 3.2. JPYSF Ameliorated Renal Pathological Injury in 5/6 Nx Rats

**Figure 2:**
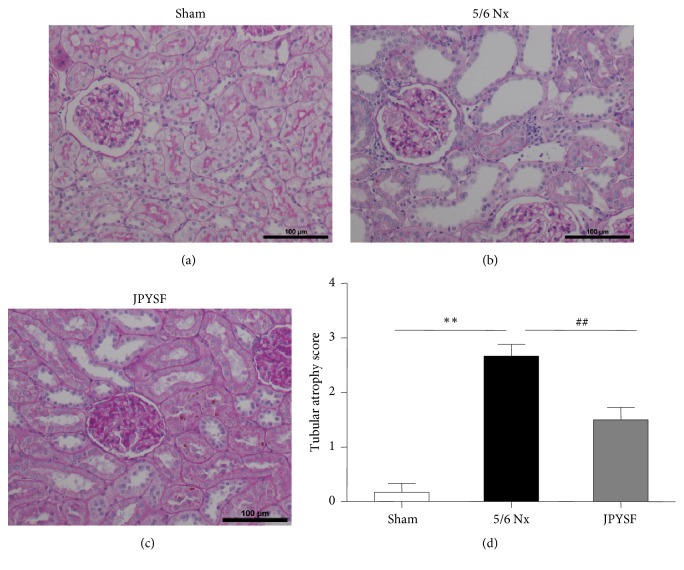
**JPYSF ameliorated tubular atrophy in 5/6 Nx rats.** PAS staining showing representative image of renal tissue from the sham group (a), the 5/6 Nx group (b), and the JPYSF group (c). All images are shown at identical magnification, ×200. (d) Tubular atrophy score. Data are presented as the means ± SEM, n=6 rats per group (^*∗∗*^*P*<0.01 compared with the sham group; ^##^*P*<0.01 compared with the 5/6 Nx group).

**Figure 3:**
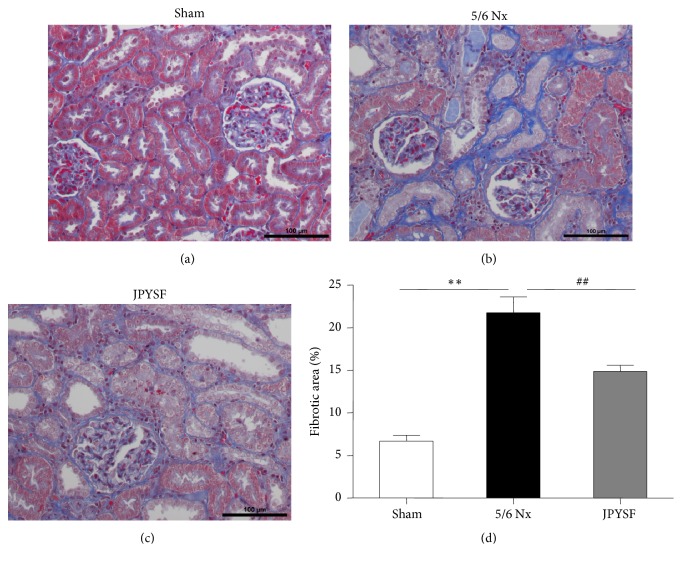
**JPYSF ameliorated interstitial fibrosis in 5/6 Nx rats.** Masson staining showing representative image of renal tissue from the sham group (a), the 5/6 Nx group (b), and the JPYSF group (c). All images are shown at identical magnification, ×200. (d) Quantitative analysis of fibrotic area. Data are presented as the means ± SEM, n=6 rats per group (^*∗∗*^*P*<0.01 compared with the sham group; ^##^*P*<0.01 compared with the 5/6 Nx group).

PAS staining displayed normal kidney structure in the sham group. In contrast, prominent tubular atrophy was observed in the 5/6 Nx group, which was further proved by quantitative analyses (*P*<0.01) (Figure 2). In Masson staining, 5/6 Nx rat showed obvious interstitial fibrosis, which was about 4 times of the sham group in quantitative measurement (*P*<0.01) (Figure 3). The treatment of JPYSF significantly ameliorated tubular atrophy and interstitial fibrosis in 5/6 Nx (Figures 2 and 3). Tubular atrophy and interstitial fibrosis are pathological characteristic of CKD and common pathway from CKD to end-stage renal disease [24]. Our data suggested that improvement of kidney function in JPYSF group may be associated with protection of kidney structure.

### 3.3. JPYSF Inhibited Renal Inflammatory Response in 5/6 Nx Rats

**Figure 4:**
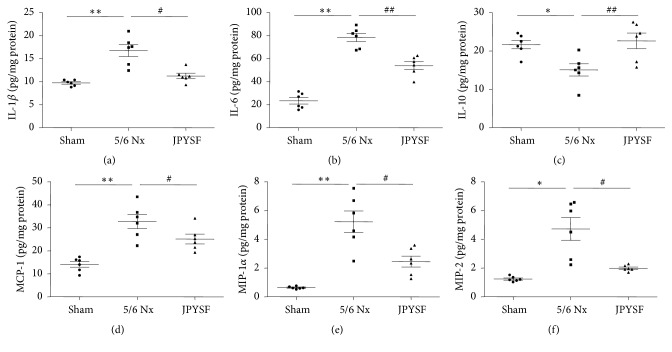
**JPYSF inhibited renal inflammatory response in 5/6 Nx rats.** The levels of multiple inflammatory mediators were measured in different groups. (a) IL-1*β*. (b) IL-6. (c) IL-10. (d) MCP-1. (e) MIP-1*α*. (f) MIP-2. Data are presented as the means ± SEM, n=6 rats per group (^*∗*^*P*<0.05, ^*∗∗*^*P*<0.01 compared with the sham group; ^#^*P*<0.05, ^##^*P*<0.01 compared with the 5/6 Nx group).

Inflammation is an important contributor to the development and progression of CKD. Then, we tested the levels of classical inflammatory mediators in the kidney. As shown in Figure 4, the levels of IL-1*β*, IL-6, MCP-1, MIP-1*α*, and MIP-2 were all upregulated in 5/6 Nx rats and could be significantly downregulated after JPYSF treatment. The anti-inflammatory cytokine IL-10 was reduced in 5/6 Nx rats. Treatment of JPYSF increased the level of IL-10. These results indicated that inflammation was activated in CKD model and could be inhibited by JPYSF.

There are many causes of inflammation in the process of CKD [25, 26]. One major factor is immune dysfunction including innate and adaptive immune systems [27]. Another major factor is believed to be associated with retention of uremic toxins, which may act as proinflammatory mediators [28]. Since inflammation is a strong risk factor of mortality in CKD patients, various interventions have been proposed to target inflammation, including lifestyle modification, pharmaceutical drug, and dialysis [29]. A wide range of biologically active compounds extracted from TCM have been proved to have anti-inflammatory effect [30]. Astragali Radix, the "sovereign medicinal" in JPYSF, has been reported to reduce IL-6 production in lipopolysaccharide- (LPS-) stimulated human amnion cells [31]. Salviae Miltiorrhizae Radix et Rhizoma, the "courier medicinal" of JPYSF, has been observed to inhibit LPS-induced MCP-1 production in RAW 264.7 cells [32]. In the present study, JPYSF inhibited proinflammatory cytokines expression and promoted anti-inflammatory cytokines expression, which may explain the renoprotective effect of JPYSF in 5/6 Nx rats.

### 3.4. JPYSF Suppressed NF-*κ*B Signaling Pathway Activation in 5/6 Nx Rats

**Figure 5:**
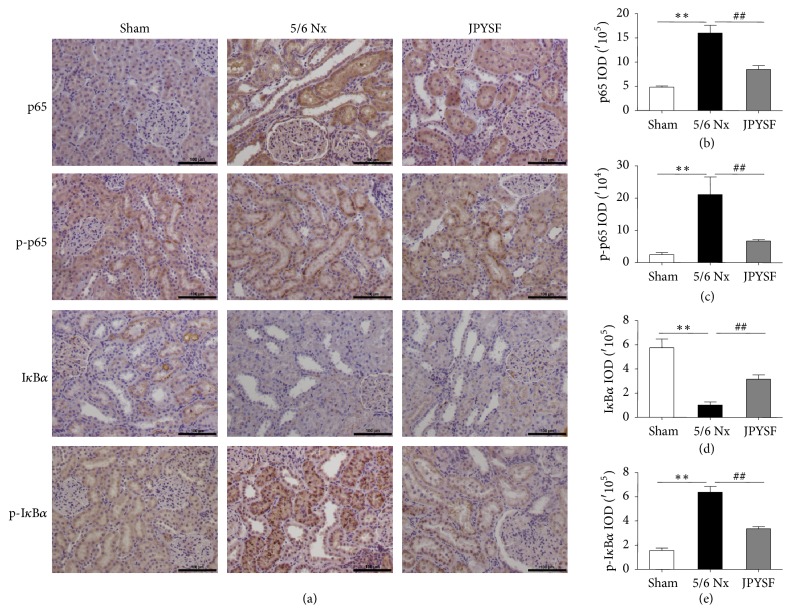
**JPYSF suppressed NF-**κ**B signaling pathway activation in 5/6 Nx rats.** Representative immunohistochemistry images (a) and quantitative analysis of p65 (b), p-p65 (c), I*κ*B*α* (d), and p-I*κ*B*α* (e) in the sham, 5/6 Nx, and JPYSF group of rat kidneys. All images are shown at identical magnification, ×200. Data are presented as the means ± SEM, n=6 rats per group (^*∗∗*^*P*<0.01 compared with the sham group; ^##^*P*<0.01 compared with the 5/6 Nx group).

We further explored the possible mechanism of anti-inflammatory effect of JPYSF in terms of NF-*κ*B signaling pathway. p65, also known as RELA, is a REL-associated protein involved in NF-*κ*B heterodimer formation, nuclear translocation, and activation. Phosphorylation of p65 is crucial posttranslational modification required for NF-*κ*B activation. In immunohistochemistry analysis, the levels of p65 and phospho-p65 (Ser 536) were obviously increased in the kidney of 5/6 Nx rats and were markedly suppressed in the JPYSF group (Figures 5(a)–5(c)). Inhibitor of *κ*B (I*κ*B) masks the nuclear localization signals of NF-*κ*B proteins and keeps them sequestered in an inactive state in the cytoplasm. NF-*κ*B-activating agents can induce the phosphorylation of I*κ*B proteins, targeting them for rapid degradation through the ubiquitin-proteasome pathway and releasing NF-*κ*B to enter the nucleus. Our data showed that 5/6 Nx rat had lower I*κ*B*α* expression but higher phospho-I*κ*B*α* (Ser 32) expression, compared with sham group. Administration of JPYSF significantly reversed the expression of I*κ*B*α* and phospho-I*κ*B*α* (Figures 5(a), 5(d), and 5(e)).

Transcription factors of the NF-*κ*B/Rel family play a pivotal role in inflammatory and immune responses. The target genes of NF-*κ*B include IL-1, IL-2, IL-6, MCP-1, tumor necrosis factor-*α*, adhesion molecules, and several other proinflammatory mediators [33]. Therefore, modulation of NF-*κ*B signal pathway is essential in ameliorating inflammation and its associated kidney disease. In the present study, JPYSF significantly inhibited NF-*κ*B signaling pathway, which was activated in 5/6 Nx rats. Similar to our results, previous studies have reported that traditional Chinese herbal decoctions containing Astragali Radix, Salviae Miltiorrhizae Radix et Rhizoma, or Atractylodis Macrocephalae Rhizoma, which are also major components of JPYSF, could modulate NF-*κ*B signaling pathway to exert anti-inflammatory effect [34–36].

## 4. Conclusions

In conclusion, this study demonstrated that JPYSF significantly retards development and progression of CKD in a 5/6 Nx rat model, which may be associated with inhibition of inflammation via NF-*κ*B signaling pathway.

## Data Availability

The data used to support the findings of this study are available from the corresponding author upon request.
